# Antifungal activity of MAF-1A peptide against *Candida albicans*

**DOI:** 10.1007/s10123-021-00159-z

**Published:** 2021-01-16

**Authors:** Rong Cheng, Qiang Xu, Fangfang Hu, Hongling Li, Bin Yang, Zonggang Duan, Kai Zhang, Jianwei Wu, Wei Li, Zhenhua Luo

**Affiliations:** 1 Department of Central Lab, Guizhou Provincial People’s Hospital, Guiyang, 550002 China; 2 NHC Key Laboratory of Pulmonary Immune-related Diseases, Guizhou Provincial People’s Hospital, Guiyang, 550002 China; 3 Department of Laboratory, Guizhou Provincial People’s Hospital, Guiyang, 550002 China; 4 Department of Cardiovascular Medicine, Affiliated Hospital of Guizhou Medical University, Guiyang, 550004 China; 5 Key and Characteristic Laboratory of Modern Pathogen Biology, Department of Human Parasitology, School of Basic Medical Sciences, Guizhou Medical University, Guiyang, 550004 China

**Keywords:** Antimicrobial peptides, Antifungal activity, MAF-1A, Fungus, *Candida albicans*

## Abstract

Invasive candidiasis is a major threat to human health, and *Candida albicans* is the most common pathogenic species responsible for this condition. The incidence of drug-resistant strains of *C*. *albicans* is rising, necessitating the development of new antifungal drugs. Antimicrobial peptides (AMPs) have recently attracted attention due to their unique ability to evade the drug resistance of microorganisms. However, the mechanism of their activity has not yet been identified. The current study analyzed the mode of action of MAF-1A by confocal microscopy, scanning electron microscopy, fluorescent staining, flow cytometry, and qRT-PCR. The results indicate that MAF-1A disrupts the cell membrane of *C*. *albicans* and enters the cell where it binds and interacts with nucleic acids. qRT-PCR demonstrated that the expression of several sterol biosynthesis–related genes in *C*. *albicans* was increased after MAF-1A treatment. Together, these findings suggest that MAF-1A exerts antifungal action by affecting both the cell membrane and intracellular components. The antifungal mechanism of MAF-1A is unique, and its identification has great research and clinical significance.

**Supplementary Information:**

The online version contains supplementary material available at 10.1007/s10123-021-00159-z.

## Introduction

Candidiasis is a fungal infectious disease caused by the genus *Candida*. Candidiasis affects the skin, mucous membranes, and other organs and is characterized by high morbidity and mortality. *Candida* is considered the third most frequent pathogen responsible for delayed infections in neonatal intensive care units (Lovero et al. 2016). Among all *Candida* species, *C*. *albicans* is the most common pathogen (Gajdacs et al. 2019; Khan et al. 2019; Papadimitriou-Olivgeris et al. 2019). Recently, an increase in the drug resistance of *C*. *albicans* has been documented, and multi-drug-resistant strains of *C*. *albicans* have been identified (Bitew and Abebaw 2018; Canela et al. 2018; Khedri et al. 2018). The threats to human health associated with the drug resistance of *Candida* infections continue to grow, necessitating the development of new and highly effective antifungal drugs.

Antimicrobial peptides (AMPs) are an important part of the natural immune systems and are widely distributed in many plant and animal species. AMPs possess an activity against bacteria, fungi, viruses, and tumor cells and exhibit immunomodulatory effects (Pasupuleti et al. 2012; van der Does et al. 2019). AMPs have many advantages over traditional antimicrobial drugs, such as low toxicity and evading drug resistance mechanisms, and are, therefore, widely considered a substitute for traditional antibiotics (Hancock and Sahl 2006; Nuti et al. 2017; Patel and Akhtar 2017).

*Musca domestica* antifungal peptide-1 (MAF-1) is a novel cationic AMP isolated from the hemolymph of *M. domestica* larvae and exhibits an excellent antifungal activity (Fu et al. 2009). MAF-1A is a linear 26-amino acid peptide fragment of the carboxy-terminal functional domain of MAF-1, containing amino acids 128–153. Our previous study demonstrated that MAF-1A exerts activity against a variety of *Candida* species, including drug-resistant strains (Zhou et al. 2016). However, the mechanism of the antifungal properties of MAF-1A remains unclear.

It has been demonstrated that AMPs act mainly by the disruption of the bacterial membrane. AMPs enter the cell membrane through electrostatic interaction, disrupting its integrity and affecting intracellular organelles. Maurya et al. (2011) documented that the antimicrobial peptides VS2 and VS3 enter into *C*. *albicans* cells, resulting in the accumulation of reactive oxygen species and cell death. Li et al. (2019) analyzed the mechanism of action of the CGA-N9 derivative of Chromogranin A (CGA) and showed that this AMP enters the cell without destroying the cell membrane, while still achieving its antimicrobial effect. However, for most AMPs, the mechanism of action is not yet identified. Therefore, the goal of the current work was to determine the mechanism of function of the antifungal peptide MAF-1A by cell staining, microscopic analysis, and molecular approaches, with the expectation of advancing the understanding of MAF-1A properties and enriching the knowledge on the novel antifungal drugs.

## Materials and methods

### Strains and culture

*C*. *albicans* standard strain ATCC10231 was maintained as previously described (Lis et al. 2010). The cells were stored at − 80 °C and resuscitated on a Sabouraud dextrose agar (SDA) (Sangon, Shanghai, China) plate for 24 h at 37 °C. Subsequently, *C*. *albicans* was maintained in Sabouraud dextrose broth (SDB) (Sangon).

### Peptide synthesis

MAF-1A and fluorescently labeled MAF-1A containing fluorescein isothiocyanate (FITC) linked at the N-terminus were synthesized by Sangon. The purity of both preparations (≥ 95%) was confirmed by high-performance liquid chromatography (HPLC). The peptides were dissolved in sterile ultrapure water at 1.5 μM and stored at − 20 °C. FITC-MAF-1A was protected from light.

### Determination of minimum inhibitory concentration values

Antifungal assays were performed according to the requirements of the Clinical and Laboratory Standards Institute (CLSI) M27-A3. Briefly, *C*. *albicans* ATCC10231 was transfected twice on the SDA plate, and the concentration was adjusted to 1 × 10^3^ to 5 × 10^3^ CFU/ml in the SDB medium. A 100 μl aliquot of the suspension was added to each well of a 96-well polypropylene microplate (NEST, Wuxi, China). MAF1A was added to achieve final concentrations ranging from 0.03 to 0.3 μM. All experiments were performed in triplicate. After incubation at 37 °C for 24 h, the minimum inhibitory concentration (MIC) value for the fungus was determined by the MTT assay as previously described (El-Mashad et al. 2012). Briefly, 10 μl of MTT solution was added to each well, and after 4 h of incubation, 100 μl of the supernatant was carefully aspirated, and an equal volume of DMSO was added. The plates were then shaken for 10 min and the optical density (OD) was measured at 570 nm using the Synergy H1 microplate reader (BioTek, Winooski, VT, USA). The lowest concentration of MAF-1A producing fungus growth inhibition ≥ 50% vs. negative control (sterile ultrapure water) corresponded to the MIC value. The MIC values for FITC-MAF-1A were determined in an identical manner, but the assays were performed in the dark. All determinations were performed in triplicate.

### Laser scanning confocal microscopy

As detailed previously (Lee et al. 2010), suspension of *C*. *albicans* at a concentration of 1.0 × 10^6^–5.0 × 10^6^ CFU/ml was incubated in the dark with FITC-MAF-1A (0.24 μM, MIC) at 37 °C for 3, 4, and 6 h. The cells were then collected, washed 3 times with sterile PBS, and mixed with 10 μl antifade medium on a glass slide. The fluorescence was examined at the excitation wavelength of 495 nm and the emission wavelength of 520 nm using a confocal laser scanning microscope Olympus FV1000 (Olympus, Tokyo, Japan).

### Transmission electron microscopy

The morphology of *C*. *albicans* after the treatment with MAF-1A was analyzed using the HITACHI H-7650 TEM (HITACHI, Tokyo, Japan). Briefly, the concentration of *C*. *albicans* ATCC10231 suspension was adjusted to 1.0 × 10^6^–5.0 × 10^6^ CFU/ml; the suspension was mixed with 0.18 μM of MAF-1A and incubated at 37 °C for 24 h. Subsequently, the cells were collected and fixed in 3% glutaraldehyde and osmium tetroxide. Fixed cells were washed with sterile PBS, dehydrated by acetone gradient, and embedded in resin. Finally, cells were stained with uranyl acetate and lead citrate and examined by transmission electron microscopy (TEM). Sterile PBS was used as a negative control, while fluconazole (0.33 μM) and 5-fluorocytosine (0.5 μM) served as positive controls.

### Cell wall staining

*C*. *albicans* suspension was mixed with the SDB medium to yield a concentration of 1.0 × 10^6^–5.0 × 10^6^ CFU/ml. The cells were then mixed with MAF-1A (final concentration, 0.18 μM) and incubated at 37 °C for 12 h. After washing 3 times with sterile PBS, smears were prepared and treated for 10 min with 10% citric acid. After washing, the specimens were stained for 1 min with 5% crystal violet, washed, dried, and observed under the microscope. PBS was used as a negative control and caspofungin (20 μg/ml) as a positive control. The fraction of cells with preserved wall integrity was calculated by counting 100 randomly selected cells. All experiments were performed in triplicate.

### Analysis of cell membrane integrity by flow cytometry

Suspension of *C*. *albicans* (9 × 10^6^ CFU/ml) was treated with MAF-1A (0.18 μM and 0.36 μM) at 37 °C for 6 h, 12 h, and 24 h. The cells were collected, washed 3 times with sterile PBS, and stained with 10 μl/ml of propidium iodide (PI) and thiazole orange (TO) for 5 min at room temperature (Cell Viability Kit, Becton Dickinson, Franklin Lakes, NJ, USA).

### Interaction of MAF-1A with nucleic acids of *C*. *albicans*

#### Prediction of the binding of MAF-1A to DNA and RNA

The presence of binding sites for MAF-1A in *C*. *albicans* DNA and RNA was identified using the protein-nucleic acid interaction prediction software BindN (http://bioinfo.ggc.org/bindn/), available at the Gene Infinity Bioinformatics website (Wang and Brown 2006).

#### Demonstration of the binding of MAF-1A to DNA and RNA by gel electrophoresis

*C*. *albicans* ATCC10231 was collected, and DNA and RNA were extracted and dissolved in the presence of varying concentrations of MAF-1A (0.09, 0.18, 0.36, 0.54, 0.72, and 0.9 μM). A sample containing 250 ng DNA was subjected to 1% agarose gel electrophoresis. PBS was used as a negative control, and sterile water as a blank control. RNA was subjected to 1% RNA formaldehyde denaturing agarose gel electrophoresis. PBS was used as a negative control, and DEPC water was used as a blank control. Images of the gels were acquired and analyzed by the WD-9473B gel imaging analyzer (Liuyi, Beijing, China).

#### Footprinting assay

*C*. *albicans* ATCC10231 was collected and DNA was extracted. A 10 μl aliquot of DNA (0.2 mg/ml) was incubated with 10 μl of MAF-1A solution (0.18 μM and 0.36 μM) at 37 °C for 1 h. Subsequently, 2 μl of the reaction mixture was combined with 5 μl of MgCl_2_/CaCl_2_ solution (10 mmol/l MgCl_2_, 5 mmol/l CaCl_2_, filtered and sterilized) and 2 μl of buffer (200 mmol/l HEPES-KOH, 50 mmol/l KCl, 4 mmol/l spermine, 0.02 mmol/l zinc acetate, 0.1 μg/ml bovine serum albumin, 10% (v/v) glycerol, and 0.5 mmol/l DTT, pH 7.9). After mixing, 1 μl of 200 U/ml DNase I solution and 5 μl stop solution (20 mmol/l EDTA, 1% (w/v) SDS, 0.2 mol/l NaCl, 125 μg/ml yeast tRNA, pH 8.0) were added sequentially, and the sample was incubated for 37 min at 37 °C. After the termination of the reaction, electrophoresis was performed on 2% agarose gel. PBS was used as a negative control (drug-free control) and sterile water as a blank control (to exclude the contamination of reagents). All experiments were performed in triplicate. The results were recorded with a gel imaging analyzer.

### Detection of MAF-1A gene expression in *C*. *albicans* by quantitative RT-PCR

RNA was extracted from MAF-1A-treated *C*. *albicans* at 12 h and 24 h, and the expression of three ERG genes (ERG5, ERG6, ERG11) involved in ergosterol biosynthesis was determined by qRT-PCR. The assay was performed using the CFX-Connect Real-Time System (Bio-Rad, Hercules, CA, USA) and the SYBR Premix Ex Taq^TM^ kit (Takara, Dalian, China). The amplification protocol followed the instructions provided by the manufacturer of the kit. Briefly, the reaction mixture (20 μl) was subjected to 40 cycles of 95 °C for 30 s, 95 °C for 5 s, and 60 °C for 30 s. Gene expression levels were calculated using the 2^-ΔΔCt^ method (Livak and Schmittgen 2001) with ACT1 as the internal reference gene. All experiments were performed in triplicate. The Student *t* test was used to calculate two-tailed *p* values, and *p* < 0.05 was considered to indicate a statistically significant difference. The primer sequences are listed in Supplementary Table S1.

## Results

### MIC values of MAF-1A and FITC-MAF-1A

MAF-1A and FITC-MAF-1A exhibited significant antifungal effects against *C*. *albicans*. The MIC value of MAF-1A was 0.18 μM and the MIC value of FITC-MAF-1A was 0.24 μM (Table 1).

**Table 1:** MIC values of the MAF-1A and FITC-MAF-1A

Concentrations	0.03 μM	0.0.6 μM	0.0.9 μM	0.12 μM	0.15 μM	0.18 μM	0.21 μM	0.24 μM	0.27 μM	0.3 μM
MAF-1A (%)	2.18 ± 0.12	7.38 ± 0.27	11.07 ± 0.11	26.80 ± 0.0.52	27.84 ± 0.55	71.13 ± 0.31	75.26 ± 1.25	85.96 ± 0.77	95.97 ± 2.15	97.98 ± 2.79
FITC-MAF-1A (%)	1.11 ± 0.09	5.08 ± 0.44	11.57 ± 1.02	12.65 ± 0.71	26.73 ± 1.02	35.88 ± 2.22	42.65 ± 0.79	66.73 ± 3.01	85.88 ± 1.63	92.06 ± 3.25

MIC was defined as the lowest concentration resulting in fungus growth inhibition ≥ 50% compared with control

### Distribution of FITC-MAF-1A in *C*. *albicans*

**Fig. 1:**
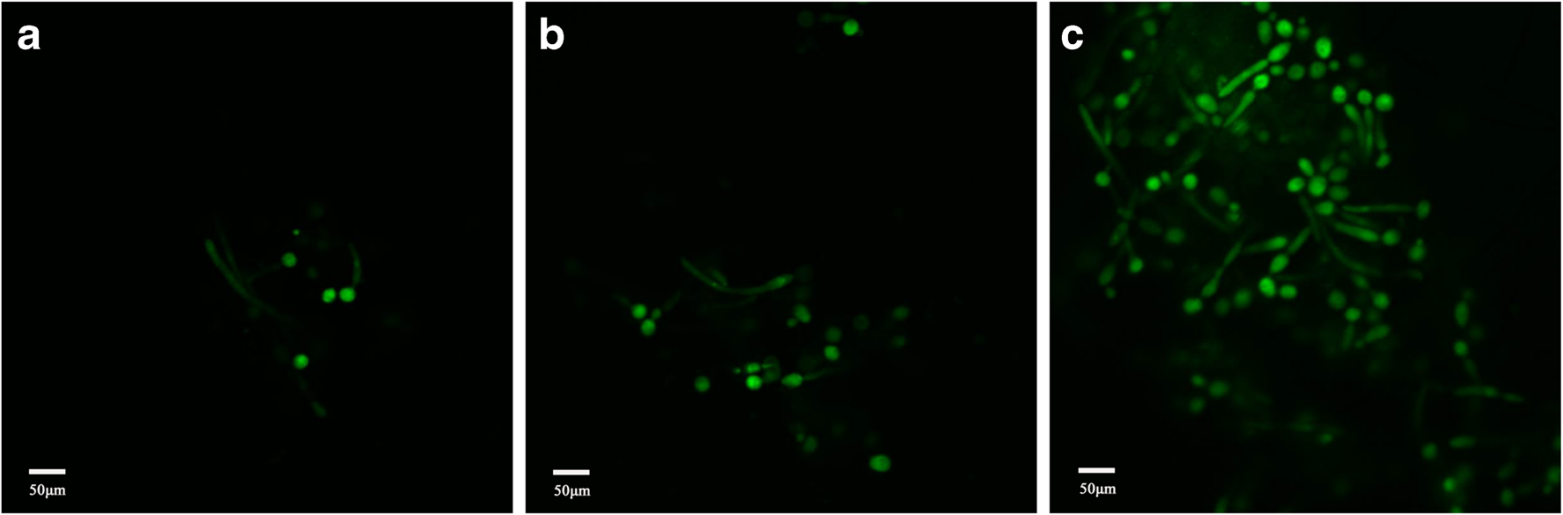
LSCM images of FITC-MAF-1A distribution in *C*. *albicans* after 3 h (**a**), 4 h (**b**), and 6 h (**c**) of incubation

Laser scanning confocal microscopy (LSCM) imaging demonstrated that the fluorescence signal was localized on the surface of *C*. *albicans* as well as intracellularly. The intensity of the signal gradually increased with the prolongation of the incubation time. The significant amount of FITC-MAF-1A accumulated within the cells indicated that MAF-1A could cross the cell wall and membrane of *C*. *albicans* and enter into the cell (Fig. 1).

### Evaluation of the effects of MAF-1A on *C*. *albicans* by TEM

**Fig. 2:**
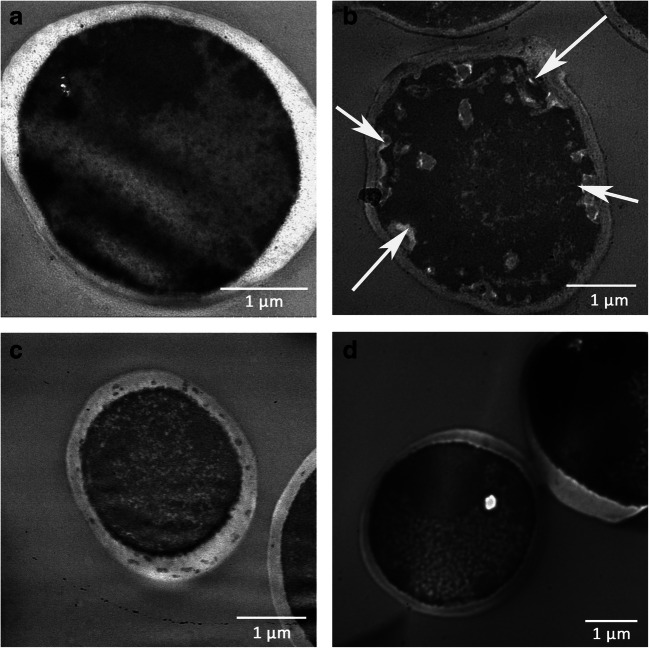
The TEM analysis of *C*. *albicans.* **a** Negative control. *C. albicans* treatment with MAF-1A (**b**), fluconazole (**c**), and 5-fluorocytosine (**d**)

The TEM imaging documented that some cells were irregular in shape after 24 h of treatment with MAF-1A. The cell wall was intact and smooth, while the cell membrane was discontinuous with multiple fractures and was partially dissolved forming gaps of different sizes. Nuclear fragmentation, poorly defined organelles, disorganized cytoplasm, multiple vacuoles, and non-uniform electron density with patchy low electron density regions were present in the MAF-1A-treated cells (Fig. 2b, arrows) but not in controls (Fig. 2a). After fluconazole treatment, some cells remained normal, round, or elliptical, the cell wall was smooth and intact, and the protoplasm exhibited high electron density. The organelles were intact, but the membrane was ruptured (Fig. 2c). In the 5-fluorocytosine-treated group, most of the cells were normal, round, or oval, and the cell wall and cell membrane were smooth and complete, but the condensation of intracellular material resulted in an increase in electron density (Fig. 2d). These findings indicate that the mechanism of the antifungal action of MAF-1A is different from that of the commonly used antifungal drugs. MAF-1A appears to act on multiple targets within *C*. *albicans* cells, suggesting the presence of a complex antifungal mechanism.

### Effect of MAF-1A on *C*. *albicans* cell wall integrity

**Fig. 3:**
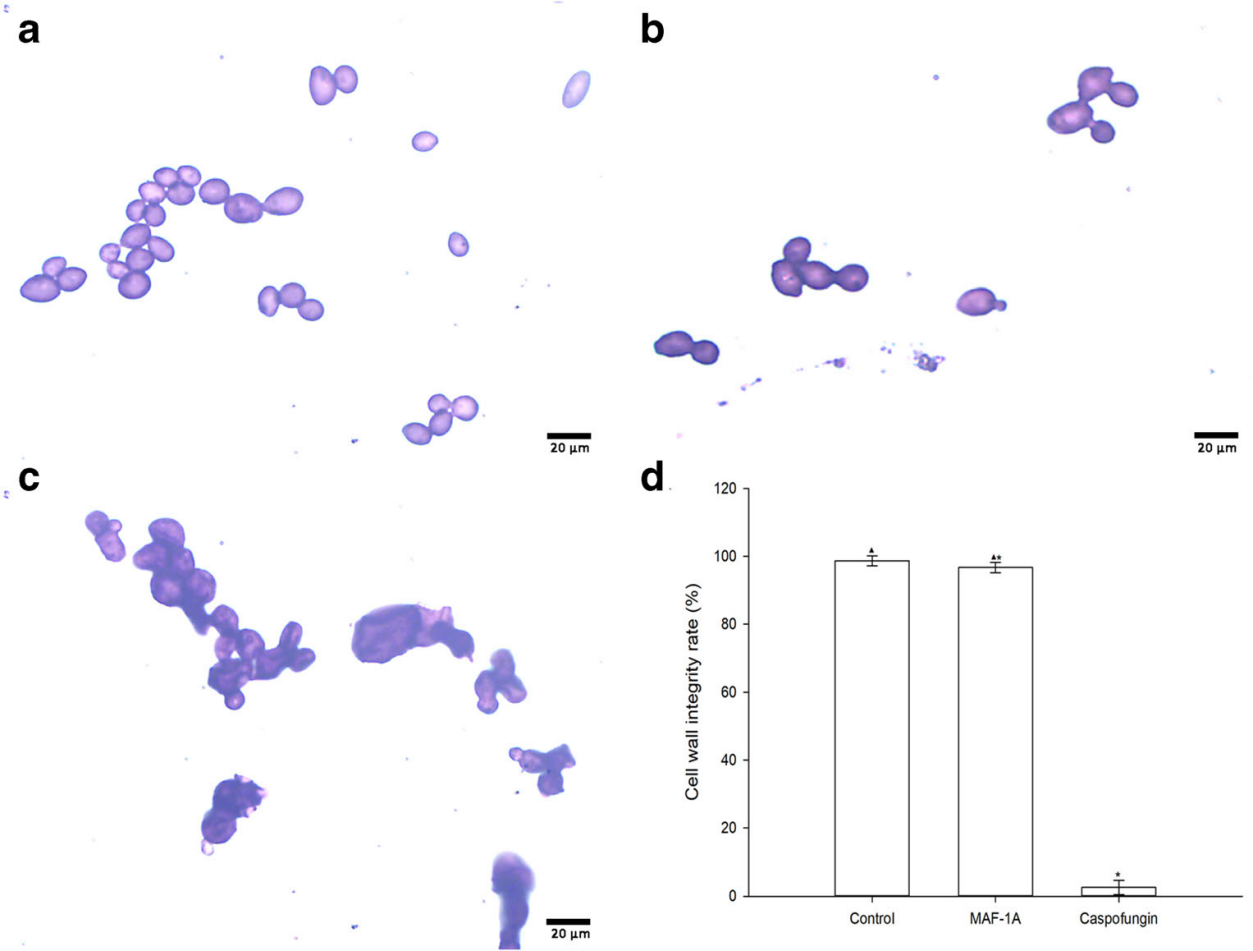
Effect of MAF-1A on *C*. *albicans* cell wall integrity. **a** Negative control (PBS). **b** MAF-1A. **c** Positive control (caspofungin). **d** Fraction of cells with the intact cell wall, *p < 0.05 vs. negative control; ^▲^*p* < 0.05 vs. caspofungin

After crystal violet staining, fungal cells with intact cell walls show purple, intact cell walls, and colorless or lavender cytoplasm. Damage to the cell wall due to drugs, death, or aging results in the entry of the dye into the cell and staining of the entire fungal cell in deep purple. *C*. *albicans* treated with MAF-1A had round or oval morphology, and an intact cell wall structure stained in purple was clearly visible outside the cell, while the cytoplasm was stained lavender. A similar pattern was noted when PBS was used as a negative control. The cells treated with caspofungin were deformed and wrinkled, their cytoplasm was stained dark purple, and the cell walls were either not easily detectable or missing. The fraction of cells with preserved wall integrity was higher in the MAF-1A group than in the caspofungin group (Fig. 3).

### Assessment of membrane integrity assessment by flow cytometry

**Fig. 4:**
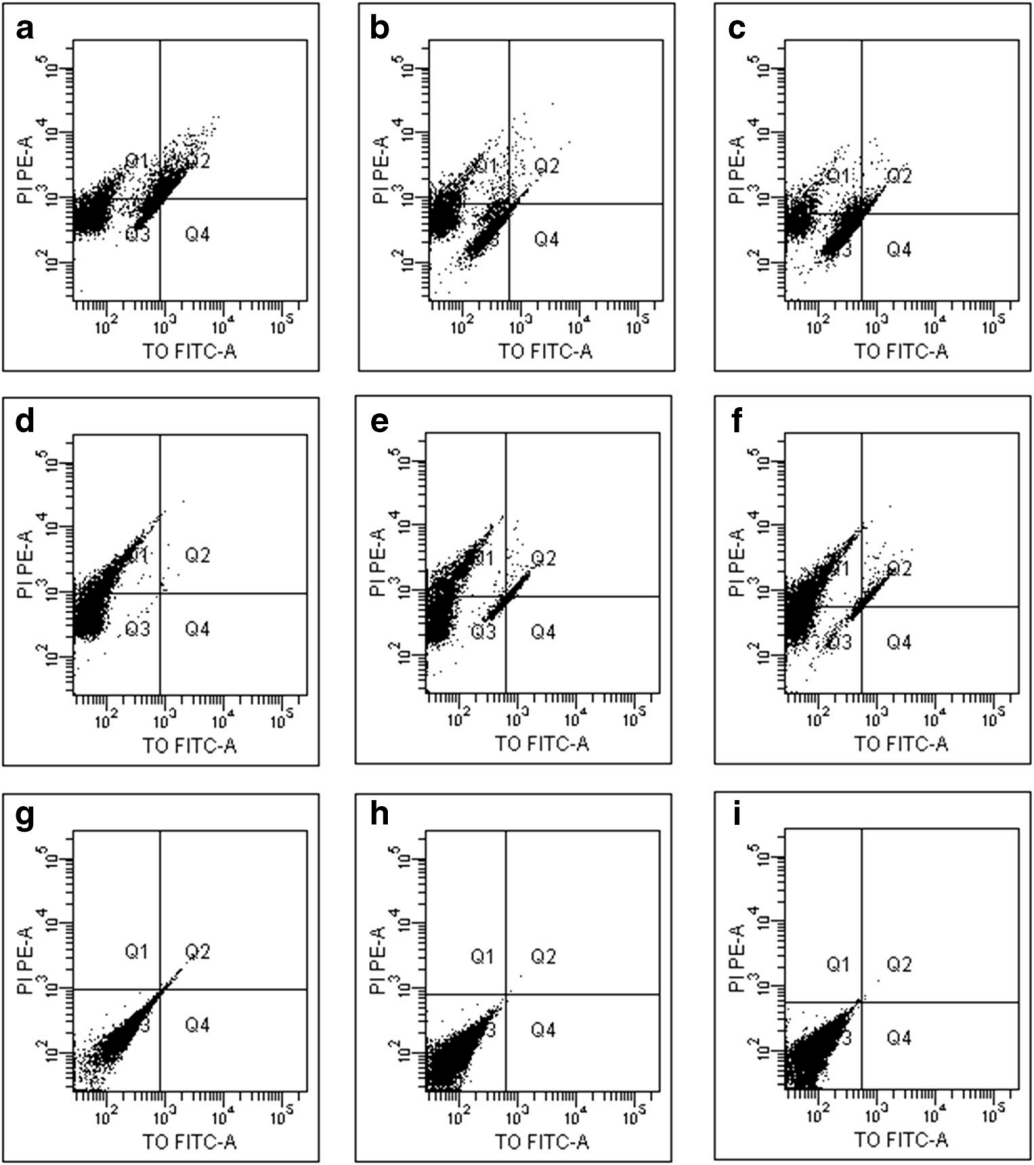
Scatter plots of PI-stained *C*. *albicans* treated with MAF-1A. **a**–**c** Treatment with 0.18 μM MAF-1A for 6, 12, and 24 h. **d**–**f** Treatment with 0.36 μM MAF-1A for 6, 12, and 24 h. **g**–**i** negative control for 6, 12, and 24 h

Flow cytometry measurements demonstrated that incubation of *C*. *albicans* with 0.18 μM (MIC) and 0.36 μM (2xMIC) of MAF-1A for 6 h, 12 h, and 24 h resulted in a time- and concentration-dependent increase in the PI signal in the cells (Fig. 4 and Table 2). Since PI becomes fluorescent upon entering the cell and binding to nucleic acids, these results indicate that MAF-1A destroys the membrane of *C*. *albicans*. The damage of the membrane becomes more severe with a higher concentration and longer presence of MAF-1A.

**Table 2:** Fraction of cells stained by PI

Time	Control (%)	0.6 mg/ml MAF-1A (%)	1.2 mg/ml MAF-1A (%)
6 h	0	8.5 ± 0.6*	12.4 ± 1.6*^,▲^
12 h	0	9.0 ± 0.3*	14.8 ± 2.1*^,▲,※^
24 h	0.1 ± 0.02	10.2 ± 1.1*^,※,§^	22.8 ± 3.2*^,▲,※,§^

*p < 0.05 vs. control

^▲^*p* < 0.05 vs. 0.6 mg/ml MAF-1A

^※^*p* < 0.05 vs. 6 h

^§^*p* < 0.05 vs. 12 h

### Interaction of MAF-1A with nucleic acids of *C*. *albicans*

#### Prediction of the binding of MAF-1A to DNA and RNA molecules

**Fig. 5:**
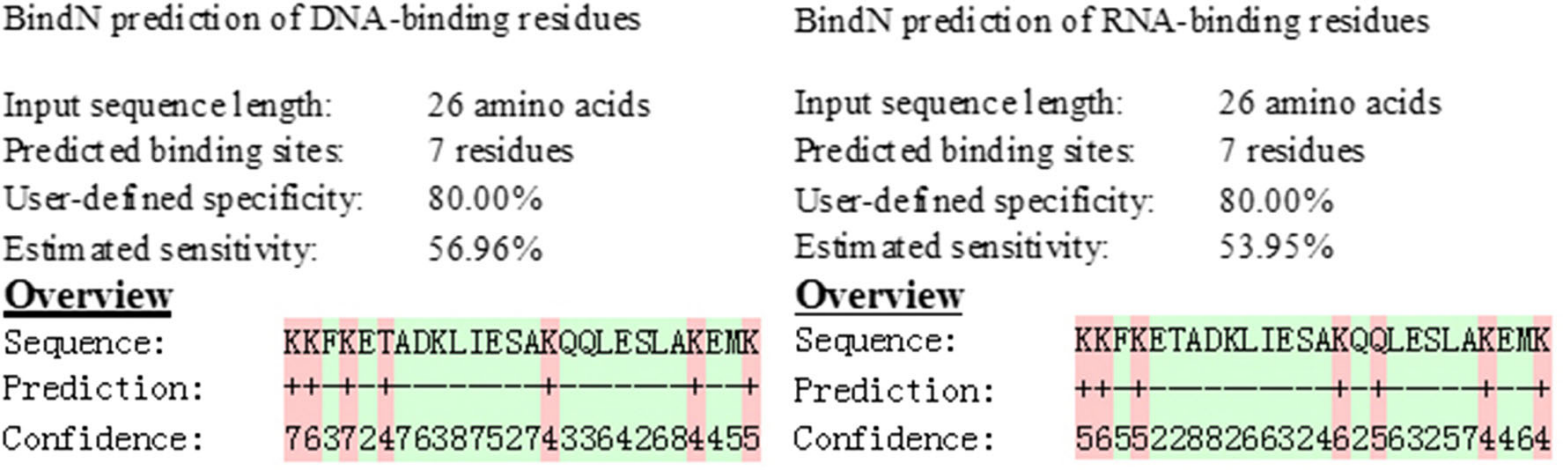
Predicted sites of interaction between MAF-1A and DNA and RNA

The BindN software predicted that the binding sensitivity of MAF-1A to DNA was 56.96%. The analysis identified 7 binding sites: K1, K2, K3, T4, K15, K23, and K26. For binding of MAF-1A to RNA, the estimated sensitivity was 53.95%, and the 7 binding sites were K1, K2, K3, K14, Q16, K23, and K26 (Fig. 5)

#### Verification of the binding of MAF-1A with *C*. *albicans* DNA and RNA

**Fig. 6:**
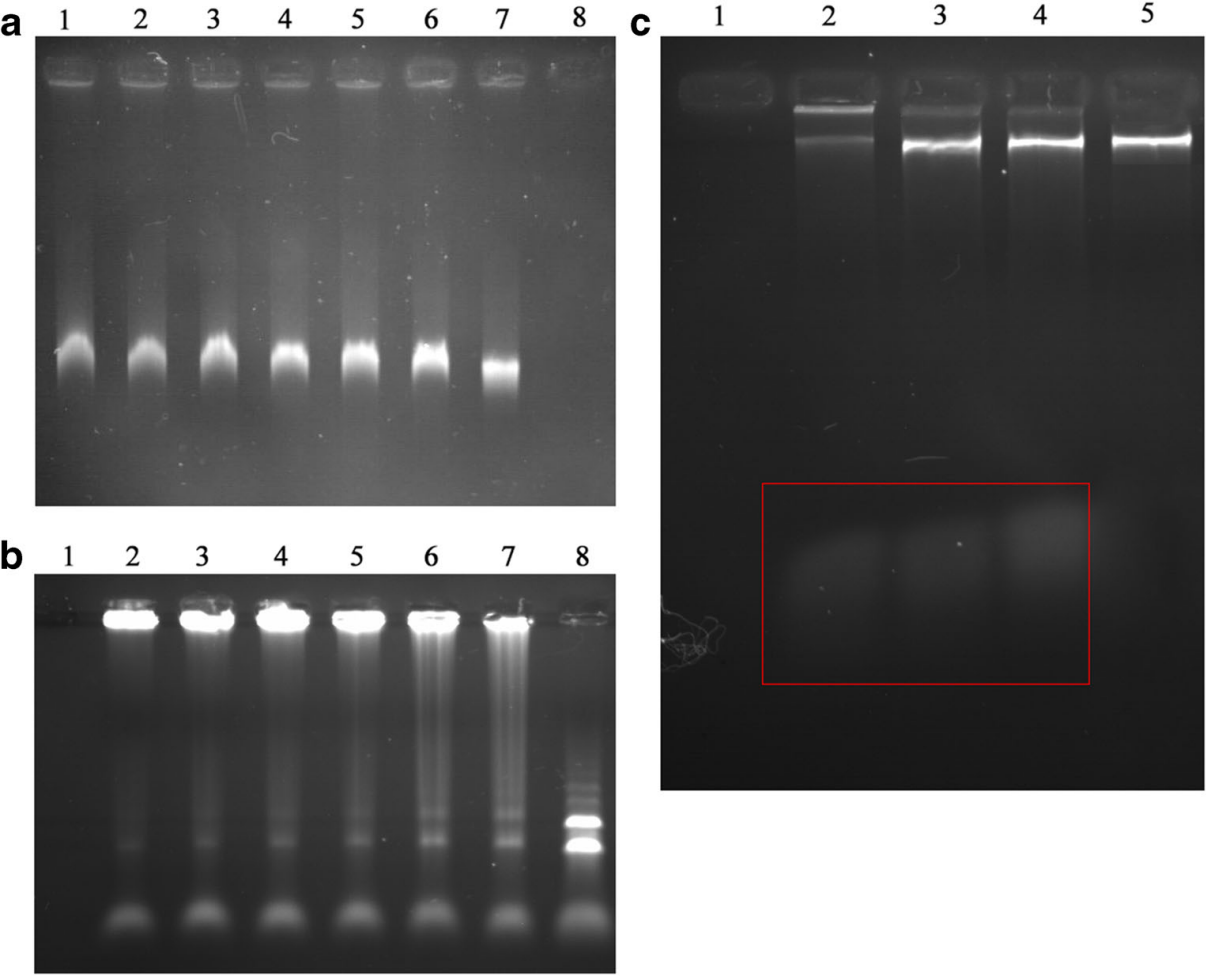
Binding of MAF-1A to *C*. *albicans* DNA and RNA. **a** Electrophoretic pattern of DNA. Lane 1, negative control; lanes 2–7, DNA treated with MAF-1A at 0.9, 0.72, 0.54, 0.36, 0.18, and 0.09 μM; lane 8, blank control. **b** Electrophoretic pattern of RNA. Lane 1, negative control; lanes 2–7, RNA treated with MAF-1A at 0.9, 0.72, 0.54, 0.36, 0.18, and 0.09 μM; lane 8, blank control. **c**, footprinting assay. Lane 1, blank control; lane 2, MAF-1A (0.36 μM) + DNA + DNase I; lane 3, MAF-1A (0.18 μM) + DNA + DNase I; lane 4, negative control, lane 5, genomic DNA

Agarose gel electrophoresis indicated that MAF-1A binds to DNA and RNA, reducing the electrophoretic mobility of these molecules and generating evident tailing. These effects were enhanced with increasing concentrations of MAF-1A. The negative control samples produced bright single bands with no tailing or dispersion (Fig. 6a, b).

#### Footprinting assay

The addition of MAF-1A to *C*. *albicans* DNA and subsequent digestion of the formed complex by DNase I produced after electrophoresis a continuous and uninterrupted gradient of small DNA fragments (Fig. 6c, marked by a red square). Some complexes of DNA and MAF-1A had a large size and did not enter the gel, remaining in the sample loading well. Importantly, the DNA fragments produced by enzyme digestion were similar in the MAF-1A and the PBS negative control groups. Thus, only non-specific small molecule fragments were generated. The absence of specific bands indicates that MAF-1A binds randomly to DNA.

### Effect of MAF-1A on the expression of sterol synthesis–related genes in *C*. *albicans*

**Fig. 7:**
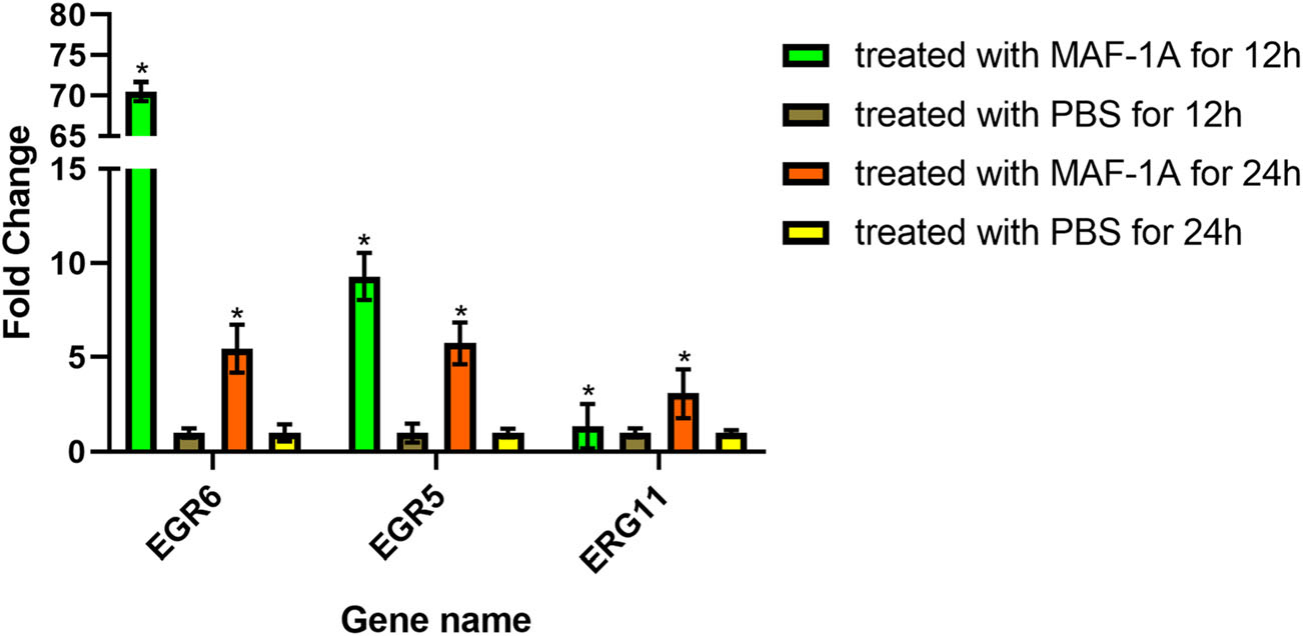
Expression of ERG6, ERG5, and ERG11 genes at the mRNA level in *C*. *albicans* treated with MAF-1A for 12 h and 24 h. *p < 0.05

The treatment of *C*. *albicans* with 0.18 μM of MAF-1A for 12 h and 24 h upregulated the expression of ERG6 (fold change 70.52, 5.45), ERG5 (fold change 9.30, 5.74), and ERG11 (fold change 1.36, 3.07) mRNA. The increases were statistically significant (*p* < 0.05). Since sterols are major components of fungal plasma membranes, this result suggests that MAF-1A may affect their structure (Fig. 7).

## Discussion

*C*. *albicans* species is the most common pathogen responsible for infections caused by the *Candida* genus. In recent years, strains of *C*. *albicans* increasingly resistant to traditional antifungal drugs have appeared, generating a significant threat to human health (Ford et al. 2015; Popp et al. 2019; Zhang and Liu 2016). Our previous work demonstrated that the antifungal peptide MAF-1A possesses an excellent activity against a variety of fungi, including drug-resistant strains (Zhenhua Luo et al. 2014). The present investigation addressed the mechanism of the antifungal action of MAF-1A. The performed experiments documented that MAF-1A disrupts the integrity of the *C*. *albicans* membrane and binds to intracellular nucleic acids, disrupting their integrity. Moreover, prolonged treatment with MAF-1A affects the expression of genes related to sterol biosynthesis. Together, the collected results indicate that the antifungal activity of MAF-1A is a complex process with multiple targets.

Our study shows that MAF-1A can enter and accumulate inside the fungus, where it interacts with intracellular structures, disrupting the normal metabolism of the fungus. Thus, the hypothesis can be raised that the key mechanism of antifungal activity of MAF-1A against *C*. *albicans* is the disruption of the cell membrane integrity. This possibility is consistent with other studies suggesting that AMPs exert antimicrobial effects mainly by destroying the cell membrane (Perez-Peinado et al. 2018; Yang et al. 2018) and the resulting leakage of cell contents leads to cell death. However, in-depth research has documented that AMPs can interact in microbial cells with macromolecules such as DNA and RNA, protein synthesis, lysosomes, and several additional intracellular targets. For example, Bac7 acts on purine metabolism and histidine kinase, LfcinB affects transcription-related activity and biosynthesis of several cellular carbohydrates, and P-Der affects catabolic pathways of several small molecules (Shah et al. 2016). Our study showed that MAF-1A binds to DNA and RNA molecules and affects the expression of EGR6, EGR6, and EGR11 genes. Thus, the current work indicates that the antifungal activity of MAF-1A is a complex process with multiple targets, which greatly reduces the possibility of drug resistance. This notion highlights the great significance of studies on MAF-1A.

The ergosterol biosynthetic pathway is a complex process involving many genes such as ERG1, ERG7, and ERG9 that are essential for cell viability in other fungi. ERG6 encodes an S-adenosylmethionine: Δ24-methyltransferase. Erg6 mutant strains possess increased resistance to polyenes, and the sensitivity to polyenes in the related fungi *Saccharomyces cerevisiae* and *C*. *albicans* requires the ergosterol biosynthetic gene ERG6 (Young et al. 2003). Our results show that ERG11, ERG3, ERG6, ERG5, and ERG25 genes were upregulated. Ergosterol is an essential component of fungal plasma membranes. The mechanism responsible for the global upregulation of ERG genes in response to azoles remains unclear. One possibility is that the depletion of ergosterol or another sterol formed late in the pathway increases global ERG expression; another argues that the accumulation of an early substrate or toxic sterol by-product induces ERG expression (Santos et al. 2007). Our research showed that the exposure of *C*. *albicans* to MAF-1A 12 h and 24 h upregulated ERG6, ERG5, and ERG11 genes, indicating that this AMP also can affect gene expression. The disordered expression of the sterol-related genes may be caused by cell stress induced by the disruption of the cell membrane structure by MAF-1A.

## Conclusion

In summary, MAF-1A has a unique mechanism of antifungal activity, which is different from traditional drugs inhibiting the growth of fungi. MAF-1A destroys cell membrane integrity and binds to nucleic acids, perturbing the gene expression in *C*. *albicans*. The involvement of multiple targets in the mechanism underlying the activity of MAF-1A decreases the likelihood of drug resistance in fungi. Thus, MAF-1A represents an important step in the development of novel antifungal AMPs.

## Supplementary Information

ESM 1 (DOCX 12 kb)
