# Supplementary material for: Antifungal activity of MAF-1A peptide against Candida albicans
Source: Int Microbiol. 2021 Jan 16;24(2):233–42. doi: 10.1007/s10123-021-00159-z (PMC8046747; doi:10.1007/s10123-021-00159-z)
Supplement: Supplementary file 1 — (DOCX 12 kb) [file 10123_2021_159_MOESM1_ESM.docx]

Table S1: The primers for qRT- PCR.

| Gene name | Sequence |
| --- | --- |
| ACT1 | F: 5’-CAAACCACTTTCAACTCCATCA-3’ |
|  | R: 5’-GAACCACCAATCCAGACAGAG-3’ |
| ERG6 | F: 5’-TGGTTGGGGTTCTTCATTCC -3’ |
|  | R: 5’-CCAGGACCACCTACACCACA -3’ |
| ERG5 | F: 5’-GATACCGTCCACCAGTCTTGA-3’ |
|  | R: 5’-TTTAGGAGCAGTGTAGGATTCAG-3’ |
| ERG11 | F: 5’-CATTTGGTGGTGGTAGACATAGA-3’ |
|  | R: 5’-AATCAGGGTCAGGCACTTTA-3’ |
| CDR1 | F: 5’-ATCCAACACCAGGGAAACTT-3’ |
|  | R: 5’-TCGCAACACCATACCTCACT-3’ |
